# State of the Patients Admitted on the Bristol Dispensary, from the 1st of October to the 1st of November, 1801

**Published:** 1801-12

**Authors:** W. D. Rolfe

**Affiliations:** Dispensary House


*State of the Patients admitted on the Bristol Dispensary, from the* 1*ft of October to the* 1*ft of November*, 1801.

| | | | |
|---|---|---|---|
| Afthenia | 5 | Abortion | 1 |
| Anafarca and Afcites | 3 | Bilious | 2 |
| Afthma | 1 | Confumption | 4 |

Dyfentery

| | | | |
|---|---|---|---|
| Dysentery - - - - - - | 21 | Puerperal Fever - - - - | 1 |
| Diarrhœa - - - - - - | 2 | Pleuritis - - - - - - - | 1 |
| Dyspepsia - - - - - - - | 1 | Rheumatism - - - - - - | 1 |
| Febris Simplex - - - - | 3 | Retrovertio Uteri - - - | 1 |
| Impostume - - - - - - | 2 | Scarlatina Anginosa - - - | 1 |
| Menorrhagia - - - - - | 1 | Typhus Fever - - - - | 40* |
| Nervous - - - - - - - | 3 | Vertigo - - - - - - - | 1 |

Dysenteries have been of late very prevalent in this city; the symptoms have been uncommonly violent, attended with great discharges of blood, and considerable pains, which have proved difficult and obstinate to cure. Astringents have by no means been beneficial; as soon as opiates have been given, tenesmus came on, with violent pains and discharges of blood. In that case I have given Magnesf. vitriolat. ℥j. and repeated it as often as the strength of the patient would admit. This proved the greatest relief; it has always brought away more or less scybala, when the pain and tenesmus have abated, and discharges of blood disappeared. After the bowels have been well evacuated, I have given the following mixture with success: ℞. Tinct. opii ʒj. Vin antim. t. ʒij. Syr. simp. ʒij. Aq. menth. pip. q. s. ut ft. mist. ℥viij. Capt. coch. iij. amp. 4tis horis.

Dysentery combined with Typhus have appeared very frequently, and have been found very tedious to cure.

W. D. ROLFE.

*Dispensary House, Nov.* 10, 1801.

---

* The Editors do not intend to oppose their opinions to those of their Correspondents; but when they observe facts asserted which contradict their uniform experience, they are tempted to ask, for the sake of information:

Can TYPHUS be so frequent at this season of the year?

Do indurated fæces REMAIN in the bowels after bilious diarrhœa?

